# Experimental models of endocrine responsive breast cancer: strengths, limitations, and use

**DOI:** 10.20517/cdr.2021.33

**Published:** 2021-07-08

**Authors:** Robert Clarke, Brandon C. Jones, Catherine M. Sevigny, Leena A. Hilakivi-Clarke, Surojeet Sengupta

**Affiliations:** 1 The Hormel Institute and Department of Biochemistry, Molecular Biology and Biophysics, University of Minnesota, Austin, MN 55912, USA.; 2 Department of Oncology, Georgetown University Medical Center, Washington, DC 20057, USA.; 3 The Jackson Laboratory, 600 Main Street, Bar Harbor, ME 04609, USA.

**Keywords:** Breast cancer, experimental models, PDX, xenografts, study design

## Abstract

Breast cancers characterized by expression of estrogen receptor-alpha; ESR1) represent approximately 70% of all new cases and comprise the largest molecular subtype of this disease. Despite this high prevalence, the number of adequate experimental models of ER+ breast cancer is relatively limited. Nonetheless, these models have proved very useful in advancing understanding of how cells respond to and resist endocrine therapies, and how the ER acts as a transcription factor to regulate cell fate signaling. We discuss the primary experimental models of ER+ breast cancer including 2D and 3D cultures of established cell lines, cell line- and patient-derived xenografts, and chemically induced rodent models, with a consideration of their respective general strengths and limitations. What can and cannot be learned easily from these models is also discussed, and some observations on how these models may be used more effectively are provided. Overall, despite their limitations, the panel of models currently available has enabled major advances in the field, and these models remain central to the ability to study mechanisms of therapy action and resistance and for hypothesis testing that would otherwise be intractable or unethical in human subjects.

## INTRODUCTION

Breast cancer is the most prevalent cancer and the second leading cause of cancer deaths after lung cancer in women^[1]^. Of the estimated > 280,000 breast cancer cases newly diagnosed in the United States annually, approximately 70% will express estrogen receptor alpha (ER; ESR1)^[2]^. For ER+ breast cancer patients, the recurrence risk after five years of adjuvant endocrine therapy currently ranges from ~40% (T_2_ N_4–9_ at diagnosis) to 10% (T_1_ N_0_)^[3,4]^. Many tumors that respond to an endocrine and/or cytotoxic therapy later recur as metastatic disease. Recurrent ER+ breast cancer is rarely cured^[2]^. Interventions that interfere with ER activation have been available for over 120 years. Unfortunately, the inability of endocrine therapies to cure all ER+ breast cancers has also been a barrier to progress since the 1890s^[5]^. Human breast cancer cells growing *in vitro* and as xenografts, chemically induced mammary tumors, and other animal models have been central to advancing our understanding of the endocrine responsiveness of ER+ breast cancer and to the development of drugs that are now in widespread use as standard of clinical care. In this review, we discuss several of the key issues associated with the use of these models in breast cancer research.

Early endocrine interventions for breast cancer patients included oophorectomy^[5]^, followed later by adrenalectomy and hypophysectomy. The first systemic intervention for ER+ breast cancer was high-dose estrogen administration, with cytotoxic chemotherapy beginning later. Unfortunately, ER+ breast cancers often exhibit a lower response rate to cytotoxic chemotherapy than other breast cancer subtypes^[6,7]^. The first antiestrogen (tamoxifen; TAM; Nolvadex^®^) was introduced in the early 1970s^[8]^. TAM remains widely used in premenopausal women but has been supplanted by aromatase inhibitors as the first-line endocrine therapy for postmenopausal women. Differences in progression-free and disease-free survival currently favor the use of the aromatase inhibitors over TAM, as well as in the patterns of side effects, and they can help to direct an optimal therapy to individual patients. However, there is no compelling evidence of any major or widespread difference in overall survival rates between TAM, a partial ER agonist, and the aromatase inhibitors that block estradiol production^[9,10]^.

The first advance beyond TAM in the context of an improvement in overall survival obtained from targeting ER was reported for the ER antagonist fulvestrant (Faslodex, Ibrance^®^), which showed increased progression-free and overall survival activity and comparable quality of life benefit when compared with an aromatase inhibitor^[9,11–13]^. Unfortunately, the optimal dosing of fulvestrant was not used in the initial clinical trials; 500 mg (not 250 mg) is now the standard dosing. Despite evidence of superiority over an aromatase inhibitor for overall survival^[14,15]^, fulvestrant (500 mg) remains largely relegated to second-line therapy for postmenopausal patients. Fulvestrant requires injection as a depot that can be painful, whereas TAM and the aromatase inhibitors are given orally. It remains to be seen whether this pattern of use will persist long into the future, or whether aromatase inhibitors will be replaced by orally active ER antagonists, several of which are actively receiving clinical evaluation^[2]^.

Most recently, combinations of an endocrine therapy (aromatase inhibitor or fulvestrant) with a CDK4/6 inhibitor have shown a significant improvement in clinical benefit relative to single agent endocrine therapy^[16,17]^. While many CDK4/6 inhibitors are under investigation, only ribociclib (Kisqali^®^) and abemaciclib (Verzenio^®^) in combination with an endocrine therapy have thus far shown any further increase in overall survival beyond that seen with single-agent endocrine therapy^[18–20]^. The efficacy of histone deacetylase inhibitors (HDAC) in combination with endocrine therapy is also being investigated in at least 14 ongoing clinical trials, although these regimens also have shown only modest improvements in clinical benefit^[21]^. Despite these advances and the notable improvements in disease-free and progression-free survival for patients with ER+ breast cancer, many patients experience distant recurrence that is eventually fatal. Clearly, there is much room for improvement. Among the most pressing issues is acquiring a greater understanding of endocrine resistance, whether *de novo* (some ER+ tumors never respond to an endocrine intervention) or acquired resistance (many ER+ breast cancers recur decades after an apparently successful treatment). Experimental models, both *in vitro* and *in vivo*, are likely to remain central tools in experimental approaches to acquiring this new knowledge.

### Why we use experimental *in vitro* and *in vivo* models

Mechanism-based cancer research requires laboratory models of the human disease^[22–24]^. All such models have limitations. The paraphrased aphorism that “*All models are wrong but some models are useful*”, attributed to the British statistician George E.P. Box (1919–2013), is not restricted to statistical models and is clearly also applicable to experimental models of ER+ breast cancer, as we explore in this review. The determination of which model(s) is sufficiently “useful” and which model(s) is not^[25]^, along with how best these models can be used^[26]^, is not necessarily trivial. Making an accurate determination is largely, but not exclusively, an issue of understanding the characteristics of the experimental model well enough to know if it is capable of allowing the investigator to test their hypothesis in a meaningful and objectively interpretable manner when used with an appropriate experimental design. Thus, the choice of model must be guided by its ability to address the question(s) at hand, rather than forcing a question upon an inadequate or inappropriate experimental model. An otherwise appropriately designed experiment forced upon a poor experimental model can produce data that may meet the expectations of the investigator yet could be misleading. Some features of this choice may be reasonably clear - testing a new antiestrogen in ER- models would be a poor choice. However, the converse assumption that testing a new endocrine intervention in any ER+ model is correct may depend on the presence or absence of ESR1 mutations (which can affect response to aromatase inhibitors), ESR2 (which can affect activity of ESR1), inherent sensitivity of the cell line selected to established antiestrogens, or other phenotypic or molecular characteristics. In this overly simple example, knowing which model(s) not to use is somewhat easier than knowing the best model(s) in which to test a new antiestrogen.

Good experimental models of ER+, endocrine-responsive breast cancer are relatively few, despite being derived from the most prevalent breast cancer subtype. Nonetheless, for many studies, whether for initial drug screening, molecular modeling, or biomarker discovery, the *in vitro* and *in vivo* models available have generated many powerful insights. Not least among those contributions is research that supported the repurposing of the failed contraceptive TAM into one of the most successful anticancer agents for any cancer, the first molecularly targeted antineoplastic agent and the first United States FDA-approved cancer chemopreventive drug^[27–29]^. Indeed, the cytotoxic, endocrine, and immunologic agents that are now used as standard of clinical care for breast cancer patients each showed safety and activity in cell culture and/or animal models at some point in their preclinical development. In this regard, the biological properties of the models appear to have been adequate to the task of early drug evaluation, despite any other cellular, genetic, and/or molecular differences among these models and apparently otherwise comparable human breast tumors.

*In vitro* studies are perhaps most commonly used when there are technical or ethical reasons that the studies cannot be done in either animals or humans. For example, drug screening or mechanistic studies that may require gene knockdown, knockout, or cDNA overexpression are often best done first *in vitro*. Such studies can allow for multiple genetic modifications and determine their effects on responsiveness to drugs, molecular signaling, or other experimental endpoints with relative technical ease, high throughput, and low cost. The costs in time and resources to generate multiple animal models with a comparable panel of genetic modifications, and multiple drug or treatment scheduling studies, can be prohibitive. Nonetheless, preclinical animal studies are often still necessary, e.g., to support advancing a candidate drug to a first-inhuman analysis. Such *in vivo* studies are generally left until sufficient data from appropriate *in vitro* experiments are available to constrain the scope and guide the design of any necessary *in vivo* experimentation. Importantly, this approach is also ethically sound because it can greatly limit the number of vertebrate animals required to test a more directed hypothesis.

An often-overlooked advantage of cell culture models, at least for testing some hypotheses, is their relative cellular homogeneity when compared with the mixed cell populations seen in some patient-derived xenograft (PDX) models. Relative homogeneity can reduce noise in some experimental designs or readouts. However, the type of heterogeneity can be an important factor^[30,31]^. For example, cellular and molecular heterogeneity are rarely synonymous. In most cell populations, some cells will be in G0 while others in G1, S, G2, or the M phase of the cell cycle, with features of their respective transcriptomes, proteomes, and metabolomes expressed differentially. If treated with a cell cycle-dependent drug, some cells will appear sensitive and some resistant. These observations provide simple examples of molecular and drug response heterogeneity in a cell population that otherwise appears to exhibit cellular homogeneity.

### General limitations of *in vitro* and *in vivo* breast cancer models

Perhaps the most common problems leveled at tumor cell lines and animal models include: (1) the high prevalence of false positives with respect to identifying agents that are effective in preclinical studies but later show limited activity in phase II or III clinical trials; (2) long-term adaptations to growth *in vitro* that may (or may not) be known to affect a relevant experimental outcome; (3) apparently poor correlations of specific genetic or other features with those evident in tissue samples from patients; (4) species differences (spontaneous, xenograft, genetically manipulated, or chemically induced mammary tumor models mostly in rodents); or (5) lack of an appropriate tumor microenvironment or supporting structure for cell lines growing in 2D culture *in vitro* and to a lesser degree cell lines growing in 3D culture or as xenografts. The extent to which any or all of these limitations are correct, relevant, or applicable to any given study is usually hypothesis specific. We address several of these issues, in part, elsewhere in this review. Here, we briefly comment on one of the more important concerns - the rate of false positives associated with drug screening using *in vitro* and *in vivo* models^[26]^.

In most cases, *in vitro* and *in vivo* experimental models are used as the first line of exploration. This use reflects, among several considerations, the low cost and high throughput when used, for example, for *in vitro* drug discovery studies. The false positive and false negative rates of current preclinical drug screening strategies are connected because, as one rate increases, the other rate will usually decrease. For hypothesis testing, investigators seek an appropriate balance in the false positive and false negative rates. For very early drug screening, a reasonable position to take is that a high false positive rate (which identifies as active some drugs that are inactive) is more acceptable than a high false negative rate (which identifies as inactive some drugs that are active). Where the most appropriate balance between false positive and false negative outcomes lies, or how this balance should be determined, is context specific and may be affected by factors unrelated to the hypothesis, such as cost, time, availability of resources, or other constraints. Later in drug development, where an agent is finally being tested in human subjects, the costs (human and financial) often dictate a balance where the likelihood of a false discovery (whether positive or negative) is as low as possible.

There are many reasons *in vitro* models and xenografts may predict poorly for subsequent clinical activity^[26]^. However, given the long and multistep process of drug development, which can exceed ten years, laying the failure of an agent in phase II or III clinical trials squarely at the feet of the experimental cell lines and animal models used in the earliest stages of this process may not be entirely warranted. It is not that more and better preclinical models and tools are not needed, as they most certainly are^[26]^ and especially for ER+ breast cancer. Rather, focusing in this area may detract from a more objective analysis to identify flaws in every step in the process that could have a major impact on the overall process. A notably challenging issue is why many drugs that meet the requirements for successful indication of safety and efficacy in phase I and II clinical trials fail to show comparable activity in phase III trials^[29,32]^. New approaches in clinical study design, such as the use of phase 0 designs^[33]^, window of opportunity trials for biomarker discovery^[34]^, and/or a more effective use of data from regional/global populations^[35]^, continue to emerge. These and other advances may yet change the standard workflows for screening and developing some agents and eventually improve the success rate for new drugs completing phase III clinical trials.

## CELL LINES AS MODELS OF ER+ BREAST CANCER

Cell lines have limitations but have been widely proven to be appropriate models for multiple aspects of breast cancer research^[36–50]^. A key consideration is an objective and informed assessment of the limitations in these models and the application of appropriate experimental designs to hypotheses that can be tested adequately within these limitations. For example, relatively few ER+ models overexpress endogenous aromatase, thus transfection with the aromatase cDNA is more widely used to create cell line models for testing drugs for aromatase inhibition^[49–51]^. Cell lines used in 2D/3D culture and as xenografts in immune-deficient rodents enable studies that are intractable in more complex models. For example, many studies in human subjects have both technical and ethical limitations. Studies in humans essentially use patients or subjects as the model, for example, patients enrolled in a clinical trial are used to predict outcomes for the population at large. Patient selection is analogous, at least in principle, to the choice of a cell line or animal model with respect to designing an experiment to test a specific hypothesis. Detailed mechanistic studies can also be difficult when there is a need to deliver a specific genetic or molecular perturbation to the cancer cell components of a PDX model tumor, and to do so with broadly comparable efficiency in all appropriate cells in the xenograft.

Relatively few human breast cancer cell lines or PDX models express a fully endocrine sensitive phenotype in that they are ER+ and/or PR+, sensitive to all endocrine therapies currently used as standard of care in the clinic, grow both *in vitro* and *in vivo*, and metastasize routinely and to the most clinically relevant sites when injected as standard mammary fat pad inoculations in the most widely used immune-compromised mouse strains. Hence, there are few parental models from which to derive isogenic models of endocrine resistance. Here, we use the commonly understood definition of isogenic as it relates to cell line variants, that is, cells known to be derived from a common parental population. This definition does not require that cells be extensively sequenced to show that they have identical (or mostly identical) genomes, which would be largely irrelevant in many cancer cell models that can acquire multiple new genetic changes quickly relative to most normal cells. Indeed, genome instability is one of the accepted hallmarks of cancer^[52,53]^. As noted below, measuring a few well-defined short tandem repeats (STR) is usually adequate to establish the genetic lineage of a cell line variant as being isogenic as the term is commonly understood.

For testing some hypotheses, the lack of a large, well-characterized series of appropriate ER+ models (particularly of the luminal A subtype) is a limitation in the field. Since choices are limited, how well any current ER+ breast cancer model(s) reflects the most relevant aspect(s) of the human disease to the hypothesis being tested remains an important consideration.

### How well do human breast cancer cell lines reflect the disease in patients?

While it has become popular in some areas to criticize established cell lines as having limited clinical relevance, such criticism often lacks an objective assessment of precisely for which characteristics of the human disease cell lines are adequate models and for which they are clearly inadequate. An inherent assumption in several such comparisons is that a single cell line, or even a panel of similar cell lines, should be a “good” reflection of what is a highly heterogeneous tumor (intratumor variability) and a highly heterogeneous disease (intertumor variability within and among currently defined molecular subtypes). Hence, a cell line may be expected to reflect some general properties shared among all tumors in the subtype of the human disease that it is believed to represent. Whether this is a reasonable assumption as such comparisons have sometimes been constructed, often in the context of genomic or transcriptomic features, may be questionable. For example, what are the most appropriate general properties, how and why were these selected, and are these properties relevant to most hypotheses that could be tested in any given cell line? Not surprisingly, cellular heterogeneous PDX models can show a “better” correlation with more features of the human disease, but whether the use of PDX models can lead to fundamentally new insights that were not already known from established cell line models remains to be determined, as is discussed elsewhere in this review.

One limitation in these general approaches or comparisons of experimental models to the human disease is that one question that is rarely asked effectively is: how well does any random patient sample (or model derived therefrom) also reflect the same characteristic(s) of the patient population from which the sample was drawn? This is essentially asking how good is any human sample as a model of the human disease. Hence, it is often not well established whether any deviation an individual cell line exhibits from a predetermined feature(s) of a heterogeneous patient population with which it is compared is within the range present across a larger population or other broadly similar populations of breast cancers. Some features of a cell line, perhaps gained (or lost) as a consequence of prolonged culture *in vitro*, may be present only in a small or undefined subgroup of patients, artifactual and not present in any samples from breast cancer patients, and/or relevant or irrelevant to the hypothesis being tested.

Some predetermined features would (or likely should) not be present in an established cell line. For example, dynamic molecular features that are essentially a reflection of changing immune cell infiltration or the presence of stromal cells that are absent in established cell line cultures will not be represented in the cell line transcriptomes. Nonetheless, at some level, it is clear that data from established cell lines maintained in 2D cell culture can exhibit significant similarities to patient samples when matched to the appropriate breast cancer subtype^[36,43–45,47]^.

The absence of a 3D culture matrix in 2D cultures, as well as of a complex extracellular tumor microenvironment in 2D or 3D *in vitro* models, has also been raised as a limitation of established cell line models growing *in vitro* in 2D. As with many other issues raised in this review, care should be taken in the context of such concerns. If the goal of the study is to explore the extrinsic effects of the tumor microenvironment, then these models will have significant limitations. However, for many experimental endpoints, the extrinsic signals from the microenvironment are received, interpreted, and executed by the intrinsic machinery of the cancer cell, whether these signals are initiated by drugs, growth factors, cytokines, or other molecules derived extrinsically. Moreover, many of the most important functions of cells, such as progressing through the cell cycle, obtaining energy from glucose, or executing apoptosis, are highly conserved - not simply between normal and cancer cells of the same species and tissue but often widely across tissues and species.

The conservation of cellular and molecular features across different species is often referred to as evolutionary parsimony, and it can explain why studying processes in quite simple species including yeast, worms, or flies can greatly advance our understanding of what happens within human cells^[54]^. Consequently, if the external (extrinsic) signals are selected, such as a drug or growth factor exposure that affects proliferation or cell survival (sensed and executed by intrinsic functions), much can be learned correctly regarding the intrinsic features that the exposure modifies from the use of 2D and 3D cultures of established cell lines. Indeed, if a human breast cancer cell line or xenograft is considered a poor model for the reasons often advanced, as noted above, the questions then arise as to what can we hope to learn about human biology from studies in fish, flies, worms, or yeast and why have we already learned so much from the study of such organisms^[55,56]^.

For many studies, the applicability of the experimental model(s) to the clinical disease is tested indirectly. For example, a biomarker or other molecular target is identified in experimental models and the strength of a correlation between the biomarker or target and a clinically meaningful event is determined in one or more patient populations. A positive outcome - a clear and compelling correlation being found - may be interpreted that both the experimental model and the predictions associated with the molecule were “correct” (assuming that neither could have arisen by chance). A negative outcome - no correlation being found - is more difficult to interpret. For example, a lack of correlation could be because the molecule was “wrong” (the experimental model was appropriate, but the analysis wrongly identified a molecule as a “correct” candidate for further study) and/or because the experimental model was “wrong” (the model could never be able to identify a “correct” candidate in the context of the hypothesis being tested).

### Basic issues in reproducibility and experimental design for *in vitro* studies

Two common challenges to executing reproducible cell line studies are linked to quality control issues, specifically infection with *Mycoplasma* species and cross-contamination with other cell lines. *Mycoplasma* is a genus of bacteria that lacks a cell wall and is often considered the simplest form of bacteria. Unfortunately, many members of this genus are resistant to common antibiotics and can be difficult to eradicate from infected cultures. Moreover, unlike contamination with other bacteria and fungi, which often turn cell culture media turbid, cell line cultures can be infected with *Mycoplasma* and show no immediately obvious signs of this infection. Cross-contamination from other infected cell lines is common and cultures can also be infected by researchers, since *Mycoplasma* are common in the human respiratory tract. Hence, infection can be prevalent; over 70% of cultures have been reported as being infected in some studies^[57]^. The consequences of *Mycoplasma* infection can be far reaching, from altering responsiveness to steroid hormones^[58]^ and gene expression profiles to affecting tumorigenicity of xenografts in immune-deficient hosts. Hence, results from *in vitro* studies with infected cells are often unreliable and/or misleading. Unlike respiratory tract *Mycoplasma*, the breast microbiota interacts with breast cancer cells and may therefore impact the effectiveness of endocrine therapies^[59,60]^, an underexplored research area.

Since eradication of *Mycoplasma* infections is often difficult, the most effective strategies are: (1) to obtain and work only with cultures known to be free of contamination; (2) routine testing of cell line stock and other cultures in the laboratory; (3) to practice effective aseptic technique during all manipulations that require retaining viable cells in culture; and (4) returning to validated cell culture stocks within a reasonably short period of time (usually a few months) to replicate experiments. Hence, cells should be obtained from reliable sources and tested as soon as they are brought into the laboratory, with frozen stocks created only from definitively *Mycoplasma*-free cultures. Fortunately, there are several excellent and cost-effective tools and services available to test for *Mycoplasma* contamination.

Cross-contamination by other cell lines is also highly problematic. As with *Mycoplasma* infection, such contamination may not be immediately obvious. The presence of other cell lines in a cancer cell culture is reflective of the presence of multiple cell types in the tumor microenvironment and may not be visible under the microscope, particularly if the cross-contamination is from another cell line of the same or a closely related cell of origin. In addition, similar to *Mycoplasma* infection, the basic growth or other characteristics of the culture may not initially appear altered, although a clear morphological change in cells is often a sign of gross cross-contamination. Data from cross-contaminated cell lines can also be unreliable and/or misleading. While the problem of cell line cross-contamination is also prevalent, there are simple methods to detect the presence of even relatively few foreign cells within a culture. For many years, the standard approach was to compare the cell line karyotype against that of the original cell line or other cultures. Reporting the karyotype of new cell lines or variants of existing lines, such as drug-resistant variants derived from a sensitive parental population, was common practice.

**Figure 1.**
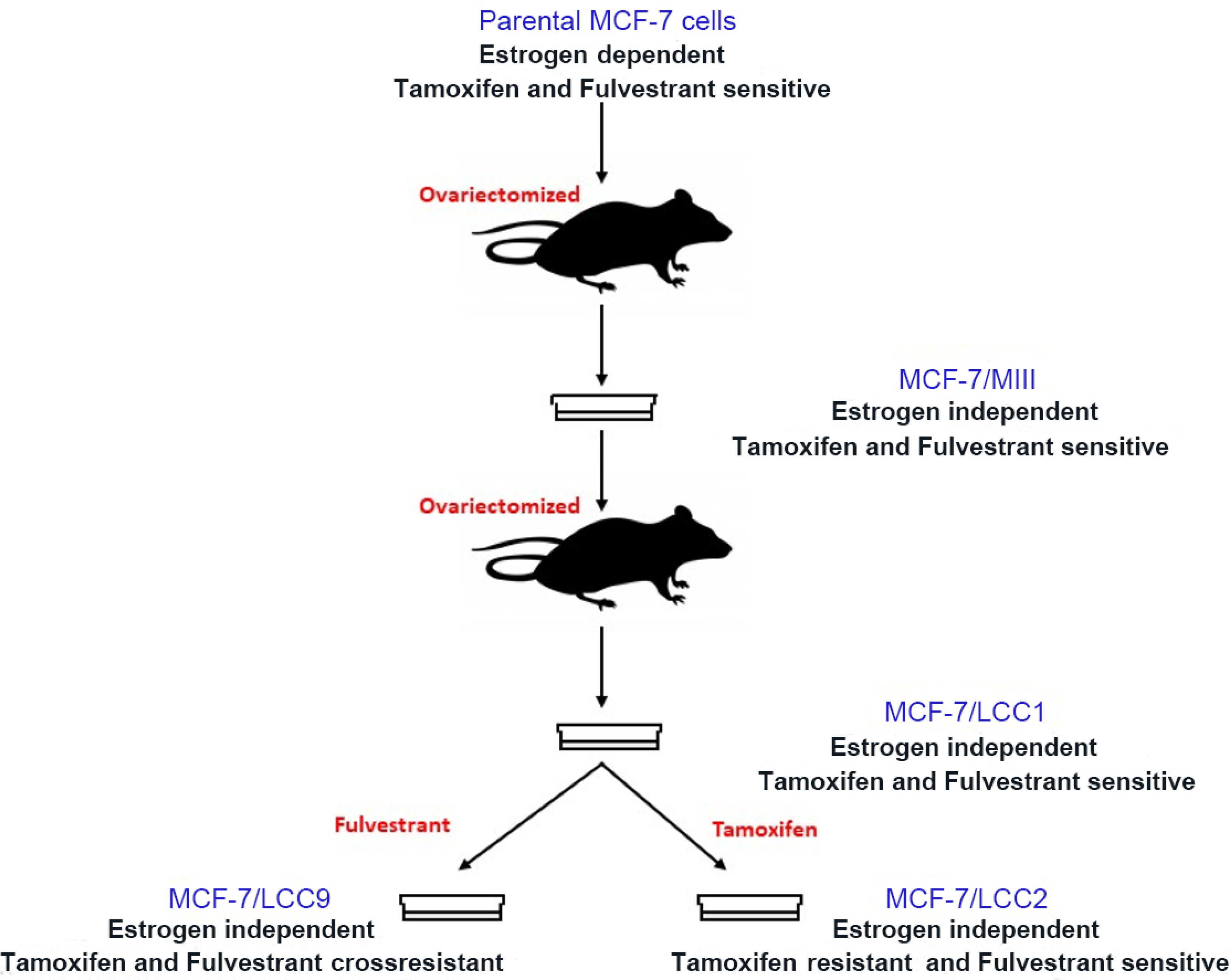
Derivation of the LCC variant series from MCF-7 parental cells.

More recently, measuring short tandem repeat (STR) patterns has proved to exhibit both high sensitivity and high specificity^[61]^ and is the current best practice for ensuring the authenticity of cell lines and the absence of cross contamination with other cells^[62]^. For example, in our hands, we analyzed 16 loci in the human genome through coamplification and multicolor detection of 15 STRs and amelogenin (gender identification) and established a genetic profile for MCF-7 cells and our MCF-7 derived cell lines [Figure 1] with a random match probability of 1 in 1.83 × 10^17^. Our other isogenic variants, as well as those we obtained for use from other laboratories including for T47D and ZR-75-1 populations, also authenticate to comparable levels of certainty. Collections of authenticated cell lines are commercially available [for example, American Type Culture Collection (ATCC); https://www.atcc.org] and from other sources^[43,63]^. Fortunately, the four basic practices noted above to reduce the risk of *Mycoplasma* infection also reduce the risk of cell line cross-contamination. Regular authentication solves one other common and related problem, knowing that the cell line being used is not an entirely different cell line^[64]^.

Experimental design is too complicated to cover all possible issues here. However, a few generic observations may be helpful to some readers. Most cell culture studies are done with cells growing on a 2D plastic surface, an approach that has proven useful for many types of studies for several decades. For some studies, a 3D culture matrix can offer a more physiologically relevant experimental context^[48,65]^. For example, 3D matrices can be prepared from ER+, human breast cancer-associated fibroblasts, or normal breast tissue (often obtained from reduction mammoplasties). During tumor progression, stromal fibroblasts can become reprogrammed to produce an extracellular matrix altered in both physical organization and physical composition. Changes include abundant collagen I, fibronectin, desmin, smooth muscle alpha-actin, TGF-β, a specific stromal isoform of the actin binding protein palladin, and other proteins that enhance tumor growth^[66]^. Studies on cell invasion often use a 3D matrix to measure the ability of cells to traverse from one side of a collagen or other matrix to the other in a Boyden chamber, Transwell, or similar apparatus^[67]^.

For many drug testing studies, using a pharmacologically based dose-response method is important, as is careful consideration of several features of experimental design^[26]^. However, bioavailability can be different *in vitro*, where the concentration of serum may affect the concentration of drug available to act. Using appropriate statistical analyses to estimate IC_50_ and IC_80_ values from dose-response data can be useful and may adjust for inherent response differences among isogenic cell lines. Recently, a growth rate inhibition endpoint has been proposed that may be less sensitive to differences in the rates of cell division among cell lines used for drug sensitivity studies^[68]^. Careful selections of the most appropriate cytotoxicity assay^[69]^ and a series of other technical and analytical parameters^[26,70]^ are critical considerations. For example, cell density can affect the rate of cell proliferation. Seeding at different cell densities can be used as a simple means to alter the proliferation rate^[71]^. However, for screening agents that may affect the cell population growth rate and/or cell cycle distribution, controlling for cell density - and thus also for the proliferative capacity of the population - is a key consideration. Many cytotoxic drugs are sensitive to the cell population growth rate and/or the proportion of actively cycling cells and can exhibit different dose-response relationships when cell populations are seeded at different densities and/or growing at different rates.

The selection of cell culture media can also affect outcomes and can be particularly important for some metabolism studies. For example, the concentration of glucose in many cell culture medium formulations is much higher than that found in normal human serum. Historically, these high concentrations were used as a matter of convenience because cancer cell lines often metabolize glucose at a high rate and can expend the glucose available so quickly that daily medium replenishment may be required. Since glucose and other key nutrients including glutamine and pyruvate are often initially present in excess in cell culture media formulations, this can affect the apparent responsiveness to agents that affect these features of cellular metabolism. Often, metabolism studies measure changes in the kinetics of substrate-product reactions governed by mass action and so may be affected when a key substrate or product is present in significant excess.

For many studies, investigators choose ER- cell lines as negative controls for ER+ cells. While often a reasonable choice, there are many cellular and molecular differences between ER- models and ER+ models than cannot be explained only by the presence or absence of functional ER. Hence, care is needed when interpreting differences between ER+ and ER- models. Additional controls that are often advisable or preferable include comparing the effects of an intervention, or a change in molecular signaling, in the presence and absence of 17β-estradiol in both estrogen responsive and unresponsive isogenic ER+ variants. Showing the ability of an effect to be blocked by an appropriate antiestrogen (often a SERD rather than a SERM) in such models and experimental designs or the loss of an effect with genetic knockdown of ER can be informative. The latter approach can be challenging because some ER+ models, whether estrogen-dependent or -independent, may not tolerate loss of ER expression for a prolonged time, at least in our hands.

### Three major ER+ human breast cancer cell lines and their variants

Breast cancer is a heterogeneous group of diseases, which is broadly classified in the clinic into three major subtypes based on the expression or absence of estrogen receptor (ERα), progesterone receptor (PR), and human epidermal growth factor receptor-2 (HER2). Among the three subtypes, the most prevalent subtype represents 70%-80% breast cancers, which is characterized by the expression of ERα and its general dependence on estrogen for proliferation. There are several models available to study the complexity of ER+ breast cancer. Since the 1970s, breast cancer cell lines have been used to replicate and investigate key clinical and molecular features of ERα-expressing breast cancers. The three most common and widely used ER+ cell lines in the field include MCF-7, T47D, and ZR-75-1^[43,72]^. These models exhibit phenotypes, genomes, methylomes, and transcriptomes that simulate many key features of ER+ human breast tumors^[36,39–41,43–48,73]^.

MCF-7, the first ER+ breast cancer cell line established and propagated *in vitro*, was isolated from the pleural effusion of a Caucasian woman with metastatic breast cancer^[74]^. MCF-7 cells express functional ERα^[75]^. Since then, the MCF-7 cell line and its numerous variants have become standard and faithful laboratory models for many *in vitro* and *in vivo* preclinical studies. Many ERα+ breast cancer patients do not respond to or become resistant to targeted therapeutic drugs such as antiestrogens and aromatase inhibitors. In an effort to identify alterations associated with this resistance, many laboratories developed MCF-7 variants that resemble clinical characteristics of endocrine responsiveness (examples are provided in Table 1).

One of the first estrogen-independent variants of MCF-7 cells was derived *in vivo* (MCF-7/MIII), by selecting the cells from MCF-7-derived tumors in ovariectomized, athymic mice that did not receive any estrogenic supplementation^[76]^. These cells were further inoculated in ovariectomized, athymic mice to form tumors in the absence of estrogen. The cells from this tumor were reestablished *in vitro* as MCF-7/LCC1 cells^[77]^ that showed a significantly reduced lag time in the appearance of tumors in athymic, ovariectomized mice as compared to MCF-7/MIII. Another cell line, MCF-7/LCC9^[78]^, was developed *in vitro* by propagating MCF-7/LCC1 cells in increasing concentrations of fulvestrant, a steroidal antiestrogen [Figure 1]. LCC9 cells were not only resistant to fulvestrant but also showed cross-resistance to tamoxifen, a non-steroidal partial antiestrogen to which the cells had not been exposed. These cell lines have been extensively used to establish clinically relevant molecular pathways responsible for endocrine resistance and to discover novel therapeutics for endocrine-resistant breast cancers.

Other laboratories have developed MCF-7 variant cell lines that were created by cultivating MCF-7 cells in the presence of antiestrogens^[38,79–82]^ or in the absence of estrogen in charcoal-stripped media for an extended period. One such cell line derived following growth in estrogen depleted medium is known as long-term estrogen-deprived (LTED) MCF-7 cells^[83]^ and another cell line is known as MCF-7:5C^[84]^. Intriguingly, both cell lines are hypersensitive to estrogen and induce cell death when treated with physiological concentrations of 17β-estradiol^[83,85]^.

The ER+ cell line T47D was first published in 1979 and, similar to MCF-7, was also established from the pleural effusion of a breast carcinoma patient^[86]^. Several distinct T47D variant cell lines have been established over time using different treatments and clone isolations. After culture in estrogenized conditions (media containing phenol red and fetal bovine serum), T47D cells were cloned to make T47D:A18^[87]^. In the same study, parental T47D cells were also cultured in estrogen-deprived conditions (phenol red-free media with dextran-coated charcoal-treated fetal bovine serum) for over one year to produce the T47D:C4 clone. While the A18 clone retains ER/PR expression and presents increased growth in response to 17β-estradiol, the C4 clone no longer expresses ER/PR and is estrogen independent. Subsequently, unlike A18, T47D:C4 is also resistant to the antiestrogens 4-hydroxytamoxifen and ICI 164,384. A similar subclone of C4 called T47D:C42 was later established, which more stably retains its loss of ER expression even after returning to estrogen-containing media conditions^[88]^. Another variant, T47D:A18/4-OHT (T47D:4HT), was produced after six months of culture with 4-hydroxytamoxifen^[89]^. The 4HT variant is also resistant to 4-OHT and is estrogen independent, despite remaining ER+. Other notable T47D variants include T47DCO, which is ER+ but estrogen-resistant^[90]^, and T47D-LTED, which was adapted from parental cells after long-term estrogen deprivation^[91]^.

In 1978, the ZR-75-1 cell line was derived from a malignant ascitic effusion of a woman with an infiltrating ductal carcinoma and treated with antiestrogen therapy^[92]^. The patient was given tamoxifen but gained little to no clinical benefit from this treatment. The isolated cell line was ER/PR+, and, similar to MCF-7 and T47D, has become a popular model of study for ER+ breast cancer. In addition, ZR-75-1 has also yielded variant cell lines. ZR-75-9a1 was produced after one year of culture with increasing concentrations of tamoxifen, becoming tamoxifen resistant and losing ER/PR expression^[93]^. These same authors also produced an estrogen-independent variant called ZR-PR-LT by growth in estrogen-free conditions for five months^[94]^. Interestingly, this variant is inhibited by estradiol and sensitive to tamoxifen but has lost ER expression^[95]^. Another more recent study produced a group of tamoxifen-resistant lines from parental cells after 8–12 months of tamoxifen treatment including ZR-75-1 Tam1 and Tam2, as well as MCF-7 Tam1, T47D Tam1 and Tam2, and BT-474 Tam1 and Tam2^[96]^. ZR-75-1 and T47D tamoxifen-resistant variants from this study possessed reduced but not eradicated ER expression. Additionally, similar to T47D-LTED, a ZR-75-LTED variant was created in another study after long-term estrogen deprivation^[91]^.

## ANIMAL MODELS OF ER+ BREAST CANCER

Generally, *in vivo* models provide more appropriate micro- and immune environments for some studies than do established or primary cells in 2D or 3D cultures. We now include some brief descriptions of *in vivo* models and their use, with particular reference to issues relevant to the use of human breast cancer xenografts. As with all models, a model’s utility is closely linked to its likely ability to provide data that can answer the questions being asked (hypotheses) and how these questions will be answered (experimental design).

### Basic issues in experimental design and reproducibility for *in vivo* studies

We describe many of the major issues in *in vivo* experimental design and analysis above^[97–100]^. While these may seem somewhat dated, the fundamental principles described therein have not changed substantively. For example, the doses and regimens for a selection of standard cytotoxic drugs can be found in Clarke^[100]^ and an introduction to the immunobiology of nude (*nu*) and severe combined immunodeficient (*scid*) mice can be found in Clarke^[99]^. The more severely immune-compromised models that often combine *scid* mutations with those in recombination activating gene-1 (*rag1*) and non-obese diabetic (*nod*) models are described elsewhere^[101]^. More recently, a community-based series of guidelines for the design and reporting of *in vivo* studies have been published in the Animal Research: Reporting of *In Vivo* Experiments (ARRIVE) guidelines^[32,102]^. Instead of reiterating many of the details from these publications, we here provide a general overview and commentary.

The issues noted above that relate to cell origin, cross-contamination, and lack of infection with *Mycoplasma* species for cells growing *in vitro* clearly also apply to *in vivo* studies with human cell line xenografts and rodent cell line allografts. Indeed, loss of tumorigenicity or metastatic capacity in a model previously known to exhibit these features when growing *in vivo* is often an indication of *Mycoplasma* infection.

Animal studies generally require power estimates to be used during the experimental design phase. Power estimates can optimize study design by ensuring that enough animals are used to ensure that the final results can be analyzed statistically and interpreted appropriately. Too few animals can produce unreliable statistical estimates and the use of too many animals is inconsistent with ethical and institutional requirements to use as few vertebrate animals as needed to test a hypothesis *in vivo*.

Reproducible and easily interpretable endpoints and visualizations, accompanied by the results of appropriate statistical analyses in statistically powered studies, are key to appreciating and understanding the data from *in vivo* studies. Unfortunately, it is not unusual to see data presented in forms that are difficult to evaluate. For example, showing tumor size only at the end of the study, rather than the entire growth curve, can be misleading or limit the ability to determine whether the effect of a drug is cytotoxic or cytostatic. Comparing only the end size ignores the repeated measures nature of tumor growth studies and can be misrepresented easily by picking only a single time for comparison. The standard assay of specific growth delay *in vivo* uses the times taken for both treated (T) and control (C) tumors to reach a predetermined size^[103]^, where growth delay = (T - C)/C and the times are taken as median values. Data from these studies are often best appreciated when the accompanying tumor growth curves are also provided. Appropriately presented growth curves can show that the starting tumor size (before initiating treatment) was the same across all groups and whether tumor size fell (at some point) below the starting tumor size (which can imply cell death and not simply growth arrest).

Generally, specific growth delay and tumor latency (time to the presence of a detectable tumor) are both explored using the Kaplan-Meier approach^[100]^, and differences among groups are studied by the log-rank test^[104]^. Repeated measures ANOVA or other tools that account for the repeated measures nature of tumor growth data and the multiple comparisons among and within groups are often used to compare tumor size at each time point across the analysis, and/or tumor doubling times are estimated and compared^[105]^. Tumor doubling times are estimated following Gompertzian transformation^[106]^, since this is usually the underlying nature of tumor growth - although many studies are completed during the apparently exponential phase of growth. Doubling times can be compared by either one-way or multivariate ANOVA^[100,105]^. Tumor incidence data (proportion of proliferating tumors/group) or proportions of metastatic lesions are usually compared using a chi-square (*χ*^2^ ) test. Multiple tumor sites in an animal are not truly independent features and can grow in a manner correlated with the host. Thus, general estimating equations^[105]^ or other approaches can be used to account for any lack of independence of tumors within animals. Measures of metastasis, particularly where the effect of an agent on the process of metastasis is the purpose of the study, can be complicated. For example, a cytotoxic drug may appear to reduce the incidence of metastases, but this may be simply a consequence of its ability to kill cells and thus be mostly unrelated to inhibiting a specific feature of the metastatic cascade. We addressed this and related issues recently^[54]^.

### Choice of immune host for cell line xenografts

The choice of an immune-deficient host is related mostly to the ability of the animal model to maintain the growth and phenotype of the recipient xenografts. The most widely used model remains the homozygous *nu* mouse, which was first reported in the late 1960s^[107]^. This model does not sustain xenografts from all cell lines and few ER+ human breast cancer cell line xenografts in nude mice appear to metastasize reproducibly in this host from routine subcutaneous or mammary fat pad inocula. As with many immune-compromised models, the animals are primarily deficient in humoral immunity but retain key features of innate immunity. Indeed, the *nu* mouse model has higher numbers of natural killer cells than its wild-type strains and retains functional macrophages and other innate immune effectors^[99]^. Nonetheless, the *nu* mouse has many advantages including ease of maintenance and use, low cost, and a large body of published data reporting the growth characteristics of many different cancer models and drug responses. Moreover, the three most widely used ER+ human breast cancer cell line models (currently MCF-7, T47D, and ZR-75-1) all form tumors in estrogen-supplemented *nu* mice, and their growth and survival (as subcutaneous or mammary fat pad inoculated xenografts) is greatly reduced by treatment with appropriate doses of TAM or fulvestrant. Thus, the use of this model remains commonplace in studies of breast cancer endocrine responsiveness. Since rodents do not express aromatase in the mammary gland, but do in the ovaries and the brain, xenograft studies to assess the effectiveness of aromatase inhibitors and the development of resistance have limitations and require careful design^[108–110]^.

The use of mammary intraductal inoculation^[111]^, rather than either subcutaneous inoculation or inoculation into the mammary fat pad (cleared or intact), recapitulates the metastatic capability of MCF-7 xenografts that was clearly present in the cells from the patient from which this cell line was derived originally^[74]^. Previously, the lack of metastatic activity *in vivo* had been widely leveled as a major criticism of the cell line and its inability to model the human disease. While likely to become much more widely used in the future, intraductal inoculation of human breast cancer cells requires specific training to perform the experiments successfully.

As the cost of other immune-deficient models continues to fall, the choice of immune-deficient hosts for xenografts is likely to extend well beyond the current widespread use of the *nu* mouse model. With the ability to produce metastases from solid intraductal tumors, rather than relying on intravenous or intracardiac inoculation to produce experimental metastases, the opportunity to study the full process of metastasis and its inhibition in ER+ human breast cancer xenografts is now more widely available. Since most breast cancer patients die from disseminated disease, this new opportunity is particularly important. Discovering antimetastatic drugs has proven difficult in other systems in which metastasis is adequately recapitulated^[112]^. While many drugs have been reported to affect metastasis, often by apparently reducing the number of metastases or delaying their appearance, the effects of drugs on these and related measures of metastasis can be confounded by general effects on tumor cell growth^[54]^. Nonetheless, a new avenue of research into whatever may (or may not) be unique about the process of metastasis in ER+ breast cancers can now be studied more effectively using cell line xenografts for which there are over 30 years of published literature as context^[54]^.

### ER+ patient derived xenografts

Since others have provided excellent reviews of breast cancer PDX models or discussed their strengths and weaknesses elsewhere^[113–117]^, we do not discuss these issues in detail here. However, similar to cell lines, but to a lesser extent, PDX models exhibit plasticity and selection bias^[116,117]^. Moreover, the extent to which any single model represents a specific feature of the entire disease can be as challenging to establish definitively for PDX models as it is for established cell lines. There is clear evidence that many individual PDX models provide a good representation of many features of the individual tumors from which each was obtained. Consequently, some investigators have claimed that PDX models are “better” representations of breast cancer than established cell lines. Given the challenges of sampling heterogeneity within a tumor, as well as the clinical, cellular, and molecular heterogeneity across tumors (within a patient, within a population, and across time)^[30,31,118]^, how well an individual PDX model represents the disease overall is largely a reflection of the representativeness of the sample from which it was derived and how well that may represent the disease. In contrast, for most established cell lines, how well these represent the individual tumors from which they were derived is not possible to know; the cell lines were derived decades previously and little if any other material remains for comparison. In general, any individual PDX or collection of PDX models may prove to be as good or as poor a representation of any specific feature to which it is compared as might any individual cell line or collection of established cell line models growing as xenografts and that shows a broadly comparable statistical correlation (or lack thereof) with the same disease feature - at least where the strength of a correlation is the measure of similarity.

As noted above, given the basic principle of evolutionary parsimony, for many hypotheses, there are likely to be more similarities between cell line xenografts and PDX models than there are differences. Thus, where a PDX model or collection of PDX models may be a more appropriate model than a comparable cell line xenograft(s) will likely be related to the nature of the hypothesis. One area that has received significant speculation is preclinical drug screening. There is a clear rationale why this could be true. PDX models retain some level of the cellular, molecular, and genetic heterogeneity that was present in the material from which they were derived, and the models have not been maintained *in vivo* or in 2D culture for a decade or more. However, whether the true false positive or false negative rates for drug discovery are better for PDX models relative to other *in vivo* screening models - as evidenced by the respective rates of validation in phase II and particularly in phase III clinical trials - remains to be firmly and (importantly) prospectively established. Clearly, it is to be greatly hoped that PDX models will prove to be consistently better predictors of clinical activity than other current models.

Despite being the largest feature of ER+ breast cancers, PDX models that accurately reflect the phenotypic characteristics of luminal A tumors are relatively rare. Unlike PDX models derived from other breast cancer subtypes, a high proportion of luminal A PDX models may not represent the primary tumor adequately^[119]^. Luminal B models are available, and these appear to be useful models in which to study important features of endocrine responsiveness^[119]^. While less common, cell lines can also be established from PDX models, as reported recently where six new cell lines were derived from pleura, primary tumor, or lymph node disease^[120]^. Whether luminal A or luminal B PDX models, or cell lines derived thereof, can identify many clinically, pharmacologically, or biologically relevant cellular or molecular responses that are not also seen in established cell line models or xenografts is unclear, but, as with predicting drug responsiveness, this is certainly to be hoped for.

### Models of ER+ rodent mammary cancer

Other than human breast cancer cell xenografts, rodent mammary tumor models mostly fall into one of four types: (1) allografts of mouse mammary tumor cells into mice; (2) chemically induced tumors in mice and rats; (3) genetically engineered mice (GEM); and (4) virus-induced tumors in mice such as mouse mammary tumor virus and polyomavirus. Induction of mammary tumors by viral infection is relatively uncommon as an experimental model, although established cell lines have been obtained from these models. Transformation with the driver mutation from polyomavirus (middle-T; PyV-mT) can produce models with mixed subtype, although additional events are required for full transformation^[121,122]^. Most GEM mammary tumor models, even the models in which mammary tumors express ER, do not respond to endocrine therapy and so have limited utility as models of functionally ER+ breast cancers. While GEM models driven by ER and/or aromatase produce preneoplastic lesions, these lesions infrequently progress to mammary carcinomas^[123]^.

The most widely used non-graft rodent mammary tumor models are those induced by chemical carcinogens. For example, the polycyclic aromatic hydrocarbon (PAH) 7,12 dimethylbenz[a]anthracene (DMBA) induces mammary tumors in some rat strains, especially in Sprague-Dawley rats. While DMBA is not found in the human environment, similar PAHs are associated with breast cancer risk in humans^[124–127]^. The rodent DMBA-induced mammary tumor model has been used for ~60 years^[128]^. Early work with this model supported the clinical development of TAM as the first molecularly targeted cancer therapy and the first FDA-approved cancer chemopreventive agent^[129]^. DMBA-treated rats develop ER+ tumors that can disappear, disappear but regrow, disappear but recur after therapy, or never respond when treated with TAM, directly mimicking patient responses^[130]^. The pathophysiology of the DMBA model directly reflects ER+ human breast tumors ^[131]^, as do their molecular features^[132,133]^ and mutational spectrum. For example, DMBA mammary tumors have mutations in *Pik3ca* and *Pten* that reflect human ER+ breast cancers^[134]^. Molecular features of autophagy, the unfolded protein response, and immune suppression are also present^[135–138]^. The alkylating agent N-nitroso-N-methylurea (NMU; MNU) is also widely used to induce ER+ mammary tumors that exhibit many features similar to the DMBA model. One notable difference between tumors induced by DMBA and NMU is the prevalence of V-Harvey ras (v-H-ras) mutations, which are present in ~80% of NMU-induced tumors but in less than 25% of DMBA-induced tumors^[98]^.

DMBA can also be used to induce ER+ mammary tumors in mice^[139,140]^, but the animals require priming (pre-treatment) with a progestin. Progestin can be given as either a single intraperitoneal injection of medroxyprogesterone acetate (MPA)^[139]^ or an implant that releases MPA throughout the study^[139]^. MPA is given when mice are six weeks of age, with DMBA subsequently administered at a dose of 1 mg via oral gavage in weeks 7–10. About 20 weeks from the first DMBA dose, 100% of the mice exhibit at least one mammary tumor. This regimen initiates mammary tumors in *C57BL/6*, *Balb/c*, *FVB*, and *DBA/2* mice. While other tumor types are rarely seen, some mice (< 10%) may develop tumors in lungs, digestive tract, skin, lymphoid tissue, or ovaries^[141]^. The mammary tumors are mostly ER+ and both HER2 and vimentin negative^[134,142]^. MPA/DMBA-initiated mouse mammary tumors are malignant^[141]^ and mostly resemble human luminal B breast cancers ^[139]^. In our hands, mammary tumors initiated by MPA/DMBA in mice do not respond to tamoxifen. Buque *et al.*^[142]^ recently reported that tamoxifen delayed the development of mammary tumors in MPA-primed, DMBA-initiated *C57BL/6* mice. However, this limits the MPA/DMBA mouse model to studying factors linked to tamoxifen-induced primary prevention of mammary tumors; the model may not be well suited to studies of those factors that drive acquired resistance. Thus, the rat remains the best of the DMBA-induced rodent models in which to investigate antiestrogen resistance and local mammary tumor recurrence after an initial endocrine treatment response.

EO771 mouse mammary tumor cells originated from a spontaneous ER+ mammary tumor in a *C57BL/6* mouse^[143]^ have been used as allografts for luminal B disease given their expression of ESR2 (ERβ), although these cells also have been classified as basal-like^[144,145]^. Since the stability and/or functionality of ER expression in EO771 tumors is inconsistently reported and basal-like tumors arise from allografted cells, EO771 has yet to be proven as a reliable and robustly responsive model in which to study ER+ breast cancers. In our hands, EO771 tumors were ESR1 negative and did not respond to physiological doses of TAM *in vitro* or *in vivo* (unpublished data). However, EO771 cells were highly responsive to the isoflavone genistein, a compound that activates both ESR1 and ESR2. These results are consistent with ESR2 being reported to be expressed in EO771 cells and activation of ESR2 inhibiting breast cancer cell growth^[145]^. Other ER+ mouse mammary tumor cell lines have also been described, such as 67NR, but whether the resulting allografted tumors are responsive to antiestrogens has not been fully established^[145]^.

## CONCLUSIONS AND FUTURE PROSPECTS

While there are adequate models for some breast cancer subtypes, the field remains somewhat constrained by the lack of a large and diverse panel of ER+ models, particularly for the luminal A phenotype but also for the luminal B phenotype. The advent of PDX models and intraductal inoculation as a site for cell line xenografts have improved the utility of *in vivo* models for testing some hypotheses. However, the general lack of a series of genetically manipulated rodent models that exhibit a consistent and reproducible ER+ and endocrine therapy responsive phenotype remains a limitation, particularly given the high prevalence of this phenotype in patients. In addition to 3D growth of established cell lines, the generation of organoids from normal and neoplastic tissue from human subjects also offers novel opportunities to study key hypotheses in a more physiologically relevant context than simple 2D culture. Such organoids can be maintained and expanded *in vitro* and can be transfected with nucleic acids, enabling several lines of mechanism-based inquiry^[146]^. Patient-derived organoids can naturally exhibit some cellular heterogeneity. One limitation is the ability to retain the mixed cellular organization and overall population phenotype over prolonged periods and multiple passages. When this limitation is reduced or eliminated, there is great potential for patient-derived organoids to become more widely available and more widely used. Another approach is to co-culture relatively homogeneous cultures such as cancer cells with different phenotypes, whether from established breast cancer cell lines or primary cultures, or co-cultured with cancer associated fibroblasts^[147,148]^. While likely less heterogeneous than PDX models and patient-derived organoid cultures, the greater homogeneity can be useful in identifying mechanistically relevant interactions among specific, predefined cell types with less noise in the data than seen with systems where cellular heterogeneity is greater.

The use of established cell lines and animal models will likely remain for the foreseeable future, particularly for those studies that are technically intractable or ethically impossible in human subjects. Hence, the need for new models that can, together, capture all of the key features of the disease will also remain. The ability of experimental models of ER+ breast cancer to lead to more major discoveries in the field will continue to require a careful choice of the most appropriate model(s) and a careful design of the experiments in which the model(s) is used.

## Figures and Tables

**Table 1.** Parental and variant ER+ breast cancer cell lines

Cell Line	ER	PR	TAM	ICI
**MCF-7**	+	+	S	S
MCF-7/MIII	+	+	S	S
MCF-7/LCC1	+	+	S	S
MCF-7/LCC9	+	+	R	R
MCF-7/5C	+	−	R	Partially R
**T47D**	+	+	S	S
T47D:A18	+	+	S	S
T47D:C4	−	−	R	R
T47D:C42	−	−	R	R
T47D:4HT	+	+	R	Partially R
T47DCO	+	+	R	R
**ZR-75-1**	+	+	S	S
ZR-75-9a1	−	−	R	R
ZR-PR-LT	−	+	S	S

ER: Estrogen receptor; PR: progesterone receptor; TAM: tamoxifen; ICI: fulvestrant; S: sensitive; R: resistant.
